# Chiral spiral cyclic twins. II. A two-parameter family of cyclic twins composed of discrete circle involute spirals

**DOI:** 10.1107/S2053273323008276

**Published:** 2023-10-31

**Authors:** Wolfgang Hornfeck

**Affiliations:** a FZU - Institute of Physics of the Czech Academy of Sciences, Na Slovance 1999/2, 182 00 Prague 8, Czech Republic; Institute of Crystallography - CNR, Bari, Italy

**Keywords:** chiral properties, spiral structures, cyclic twins

## Abstract

A mathematical toy model of chiral spiral cyclic twins is presented, describing a family of deterministically generated aperiodic point sets. Its individual members depend solely on a chosen pair of integer parameters, a modulus *m* and a multiplier μ. By means of their specific parameterization they comprise local features of both periodic and aperiodic crystals. In particular, chiral spiral cyclic twins are composed of discrete variants of continuous curves known as circle involutes, each discrete spiral being generated from an integer inclination sequence. The geometry of circle involutes does not only provide for a constant orthogonal separation distance between adjacent spiral branches but also yields an approximate delineation of the intrinsically periodic twin domains as well as a single aperiodic core domain interconnecting them. Apart from its mathematical description and analysis, *e.g.* concerning its circle packing densities, the toy model is studied in association with the crystallography and crystal chemistry of α-uranium and CrB-type crystal structures.

## Abstract 2

A mathematical toy model of chiral spiral cyclic twins is presented, describing a family of deterministically generated aperiodic point sets. Its individual members depend solely on a chosen pair of integer parameters, a modulus *m* and a multiplier μ, comprising local features of both periodic and aperiodic crystals. Chiral spiral cyclic twins are composed of discrete variants of continuous curves known as circle involutes.

## 1. Introduction

The most powerful virtues of science are found in the trinity of abstraction, idealization and generalization. This is particularly true for the exact sciences such as chemistry and physics, or the structural sciences such as crystallography and mathematics. Toy models are practical examples (Reutlinger *et al.*, 2018 ▸).

The toy model of this work arose from the experimental observation of the nucleation and growth of macroscopic tenfold twins from undercooled melts of the binary intermetallic compound NiZr. Here, abstraction in combination with idealization led to the construction of an atomistic model, and its confirmation with respect to the structure of the twin boundaries by high-angle annular dark-field scanning transmission electron microscopy (Hornfeck *et al.*, 2018 ▸). Forming the basis of an extensive theoretical study into tenfold twins of the CrB structure type (Hornfeck, 2018 ▸), this has been generalized into the concept of chiral spiral cyclic twins in Part I of this work (Hornfeck, 2022 ▸).

One aspect to consider is the dichotomy between a discrete and a continuous model, chiral spiral cyclic twins being examples of the former and circle involutes being examples of the latter. Circle involutes feature prominently in Nature, occurring, for instance, in excitable biological media, such as heart tissue (Wiener & Rosenblueth, 1946 ▸), as well as in chemical reaction–diffusion systems, such as in the Belousov–Zhabotinsky reaction (Winfree, 1972 ▸, 1974 ▸; Lázár *et al.*, 1995 ▸). Such spiral waves can occur in a segmented pattern (Vanag & Epstein, 2003 ▸), thus resembling discrete variants of the continuous curves. Discrete examples are also found in biological reaction–diffusion systems, such as in Fibonacci spiral phyllotaxis (Pennybacker & Newell, 2013 ▸). Occasionally, two-dimensional spirals can extend into three-dimensional helices, such as in the naturally discrete hives of certain bees (Cardoso *et al.*, 2020 ▸).

It is tempting to envisage their occurrence in crystal growth, where one well known growth mechanism is based on the existence of screw dislocations and associated growth spirals (Woodruff, 2015 ▸); for a theoretical treatment of polygonal spirals see Ishiwata & Ohtsuka (2019 ▸). Discrete variants of circle involutes connect these continuous growth models observed in macroscopic crystals with the existence of a necessarily discrete growth mechanism present at the atomic scale. In particular, discrete involutes offer a natural way of multiple twin growth setting in straight after the primordial nucleation stage, modelling the genesis of growth twins (Senechal, 1980 ▸).

Regarding their technological importance, due to their special geometric properties, continuous circle involutes are widely used in the design of gears (Colbourne, 1987 ▸) and scroll compressors (Gravesen & Henriksen, 2001 ▸; Liu *et al.*, 2010 ▸), yet also in something as different and unique as the design of the core of the Oak Ridge High Flux Isotope Reactor (Chandler *et al.*, 2020 ▸). Discrete variants of circle involutes are used for discrete sampling tasks, although often the closely related Archimedean spirals are utilized for this purpose. Examples are spiral grids in geophysical studies (Hüttig & Stemmer, 2008 ▸), spiral trajectories for more effective *k*-space sampling in magnetic resonance imaging (Block & Frahm, 2005 ▸; Delattre *et al.*, 2010 ▸) or optimized spiral scan paths in scanning transmission electron microscopy (Sang *et al.*, 2016 ▸), to name just a few.

Apart from these actual applications of continuous circle involutes, and the potential applications of their discrete counterparts, our toy model is also an example of an aperiodic point set (precisely, a two-parameter family of such sets). Similar to quasicrystals, chiral spiral cyclic twins distinguish themselves from other patterns by the extraordinary degree of spatial correlation existing between their constituent points. Despite lacking the periodicity of classical crystals, they possess their own regularity. Notably, aperiodic crystals featuring spiral lattices seem to be more common in biology, especially in phyllotaxis (Maciá, 2022 ▸), while aperiodic quasicrystals seem to prevail in the field of hard and soft condensed matter science. An exception are the non-periodic spiral tilings constructed by Winkler (2019 ▸), obtained from the repeated reflection of a single polygonal motif across one of its edges, according to a given edge sequence.

## 2. Generalized model

### 2.1. Mathematical description

In the following parameterization, 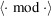 , 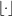 and ⌈·⌉ denote the integer modulo, floor and ceiling functions, respectively.

The mathematical model for a family of chiral spiral cyclic twins, with each member parameterized by two integers *m* and μ, is based on the use of the complex *m*th roots of unity, 

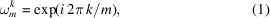


*m* in number, and their combination according to either 

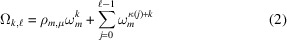

or 

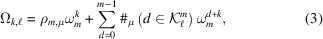

with the resulting Cartesian coordinates as two-dimensional points, 

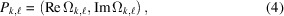

suitably indexed as the ℓth spiral node of the *k*th spiral branch, with the indexing starting at an index value of zero. The chiral spiral cyclic twin structure is then the set 

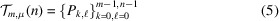

containing *m* × *n* of these points. In the following, mostly the abstract type of chiral spiral cyclic twin is referred to, being represented by the shorthand notation (*m*, μ) without specifying the number of points *n* in a single spiral branch.

Here, 

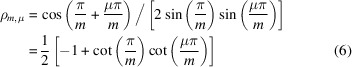

denotes the circumradius of the innermost *m*-gon, while 

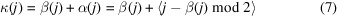

denotes an integer inclination function, encoding the coordinate evolution of the points within a single spiral branch.

For the sake of clarity, shorthand symbols are used in the following for the integer functions mentioned above, with all of them eventually depending on the parameter μ and the variable *j* only.

Now, in a corresponding point of view, the integer inclination *function* can be used to define two associated integer inclination *sequences* of length ℓ: (i) 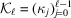 and (ii) 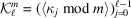 . Here, κ_*j*_ = κ(*j*). Both sequences differ only by the (non-)appearance of the modulo function, 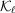 being the non-modular and 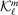 being the modular one (Appendices *A*1 and *A*2).

The two inclination sequences are interesting in themselves. In particular, 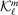 can be used to derive the parameterization given in equation (3) (Appendix *A*2). Here, 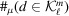 denotes a μ-dependent counting function, counting the frequency of occurrence of the number *d* in the sequence 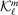 of length ℓ.

**Figure 1:**
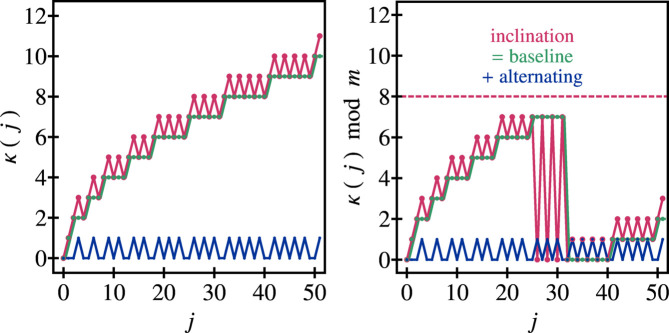
Construction of the integer inclination function κ(*j*) (red) from an integer baseline function β(*j*) (green) and an integer alternating function α(*j*) (blue). Shown are the respective functions for the case (*m*, μ) = (8, 2), (left) before and (right) after reduction modulo *m* (dashed red line).

The inclination function is itself described by an integer baseline function β in combination with an integer alternating function α. While the latter is, in fact, expressible by means of the former [equation (7)], the former is generated, in a descriptive way, by the comparatively simple rule 

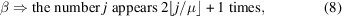

in which ‘appears’ refers to the frequency of occurrence of the number in the baseline sequence before reduction modulo *m* (Fig. 1 ▸, and Fig. 13 in Appendix *A*2).

The names chosen for these integer functions should become obvious from their respective function graphs (Fig. 1 ▸). Note that ‘alternating’ in the context of this work exclusively refers to the change between function values of zero and unity, and not to the concept of alternating series in mathematics, in which ‘alternating’ refers to a sign change occurring between consecutive terms. Note also that ‘alternating’ in the context of this work should not be understood in the strict sense of meaning perfectly alternating, but instead as predominantly alternating, since it is particularly the deviations from a perfectly alternating sequence of zeroes and ones that are essential.

Now, the rule stated in equation (8) can be expressed algebraically by means of a floor function taking a piecewise linear function as its argument, namely as 

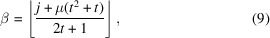

of an independent variable *j* and parameter *t* which itself, as it turns out, depends on *j* and μ:

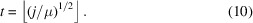

In fact, the baseline function as given in equation (9) has an extended definition as 

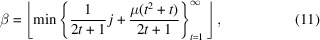

highlighting its piecewise composition from a one-parameter family of straight lines. Exactly which value of the parameter *t* yields the minimum of β within a given interval itself depends on the parameter μ and the variable *j*, in that together they define the intervals between supporting points, μ*t* ^2^ ≤ *j* < μ(*t* + 1)^2^, from which *t* ≤ (*j*/μ)^1/2^ and equation (10) follow.

This approach of creating an integer sequence of a certain preset pattern by means of taking the floor of a piecewise linear function is rather general, and due to its more systematic manner clearly superior to the previously found empirical generating formulas.

In particular, the rule for β [equation (8)] clearly determines the admissible values for μ. Since, according to the rule, μ appears in the denominator of the floor function’s argument, it cannot be zero, and since the rule counts the occurrences of a number *j*, it cannot be negative. Thus, the minimal value for μ is determined to be μ_min_ = 1.

Moreover, the rule for β determines the odd-valued lengths 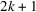 = 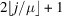 of perfectly alternating binary sub­sequences 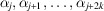 = 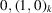 of the alternating sequence α, with μ counting the number of their consecutive repeats (Fig. 7, and Fig. 13 in Appendix *A*2). Here, the subsequences are meant to be maximal and only ever include consecutive elements of the sequence. As a consequence, the number of leading zeroes is given as μ + 1, geometrically translating into the same number of consecutive unit-distance constant inclination angle steps (φ = ±2π/*m*) occurring in the same relative direction at the beginning of a single spiral branch’s evolution. Since after *m* steps the spiral intersects itself at its starting node, a principle of self-avoidance limits the values of μ according to the inequality μ + 1 ≤ *m* − 1, resulting in μ being at most μ = *m* − 2. Admissible values for μ are curtailed even more by the observation that ρ_*m*,μ_ > 0 only holds true for 2μ + 2 < *m*, or, equivalently, μ < (*m* − 2)/2. Since μ is an integer, 

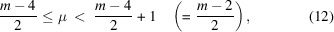

and from the definition of the ceiling function μ_max_ = ⌈(*m* − 4)/2⌉ follows. The minimal value for the twin modulus is therefore *m*_min_ = 5, coinciding with the first inclined spiral branch segment being oriented in the forward semicircle. Thus, the existence of a non-vanishing circumradius ρ_*m*,μ_ effectively prohibits a spiral’s self-intersection at its starting node.

### 2.2. Methodological derivation

**Figure 2:**
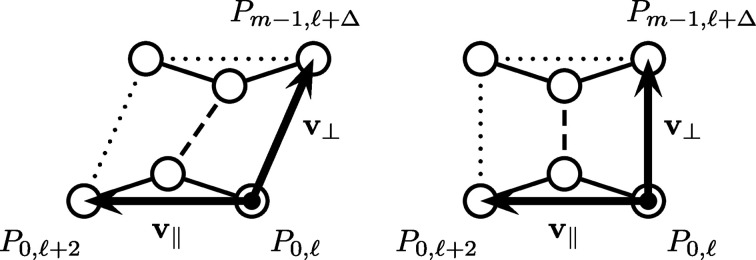
Geometric sketches illustrating the condition for the derivation of ρ_*m*,μ_. Shown are three spiral nodes each of two adjacent spiral branches, forming a chevron-like pattern 〉〈 at the centre of each unit cell within a twin domain (Fig. 8). The left-hand side illustrates the general, non-ideal, case, while the right-hand side illustrates the special, ideal, case, in which the vectors **v**_∥_ and **v**_⊥_ are perpendicular, thereby fixing the ideal value of ρ_*m*,μ_ as a function of the parameters *m* and μ.

The following is a short interlude describing the construction of the general model, in particular regarding the derivation of the ρ_*m*,μ_ function. Some further, more general, remarks are given in Appendix *B* [App appb]. The essential idea is sketched in Fig. 2 ▸.

The key insight was to demand that a part of the geometry of the chiral spiral cyclic twin be fixed in an ideal geometric motif. Such a motif is found in the centre of all unit cells within the twin domains, irrespective of the choice taken for the value of μ (Fig. 8). This is the chevron-like motif 〉〈 based on the alternating inclination taking place along each spiral branch within the twin domains. This alternating inclination defines a direction, which can be expressed algebraically by a vector **v**_∥_ running parallel along the spiral branch. Now, another vector **v**_⊥_ can be defined, starting from the same base point on one selected spiral branch, yet connecting to an endpoint on an adjacent spiral branch. In particular, the vectors are parameterized as 

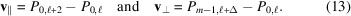

Here, Δ defines a coordinate shift yet to be determined. By visual inspection of a few patterns the following values for the parameters ℓ and Δ could be found: ℓ = μ − 1 and Δ = 2. Assuming their validity in all cases, one obtains 

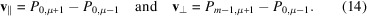

The condition to solve for is then that the pair of vectors **v**_∥_ and **v**_⊥_ be perpendicular, *i.e.* their scalar product vanishes: 〈**v**_∥_, **v**_⊥_〉 = 0. This was performed using the computer algebra system *Mathematica* (Wolfram, 2013 ▸), including subsequent steps of manual simplifications, until the following solution was found: 

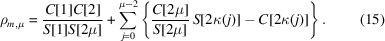

Here, 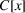 = 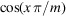 and 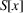 = 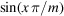 are abbreviated notations for the cosine and sine functions, respectively, of the given arguments *x*. This can be further simplified, namely to the form stated in equation (6), by observing that the first μ + 2 numbers of any inclination sequence are given by the consecutive non-negative integers starting from zero, and since the summation proceeds within a smaller index interval, from *j* = 0 to *j* = μ − 2, it is always safe to replace the inclination function just with its index: κ(*j*) = *j*.

## 3. Graphical depiction of the general model

### 3.1. Illustrations of the general model

**Figure 3:**
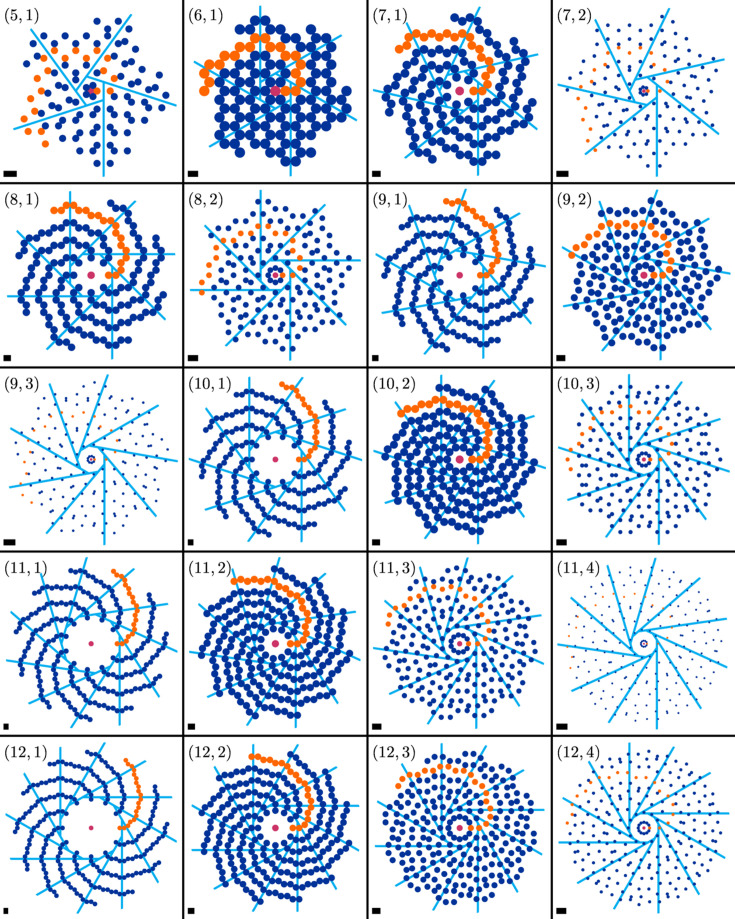
Chiral spiral cyclic twins of type (*m*, μ) (shown in the top left of each panel) up to *m* = 12 inclusive. Patterns are plotted to varying scale and the scale bars (bottom left of each panel) show one unit distance. A single spiral branch of *n* = 20 nodes is highlighted by orange discs, with a red disc marking the origin. Lines in cyan mark the twin boundaries.

Fig. 3 ▸ shows all chiral spiral cyclic twins of type (*m*, μ) for up to *m* = 12. Each spiral branch shown consists of *n* = 20 nodes, with the total number of nodes given as *m* × *n*. The graphs are brought to the same scale, thereby facilitating a better overview, while obscuring their actual extension, which expands with increasing values of *m* (mainly due to the increase in the values of ρ_*m*, μ_) and shrinks with increasing values of μ. However, the absolute scale can easily be inferred, since the distance between consecutive nodes in a single spiral branch is equal to unity (for easier comparison, scale bars of unit distance have been added). Furthermore, for any fixed value of *m* and increasing value of μ one observes a change in the pattern of closest contacts between nodes, first located along each spiral branch (purely intraspiral), later switching to be among adjacent spiral branches (purely interspiral) and sometimes including the mixed case, namely for parameter pairs following the relation *m* = 4μ + 2. In the purely interspiral case the radii of the discs are adjusted in order to prevent their overlapping.

### 3.2. Decorations of the general model

The general model as described above can be enriched by highlighting subsets of nodes according to some numerical criterion defined with respect to some of the model’s parameters. This is in fact a great advantage of the chosen parameterization. The parameters to be used can simply be the spiral’s intrinsic coordinates, *k* for the spiral’s branches and ℓ for the spiral’s nodes. Alternatively, they can be one of the integer sequence-based functions used in the parameterization, such as the one describing the alternating sequence α, the baseline sequence β or the inclination sequence κ. Eventually, the parameter *t* can also be chosen for this purpose.

The criteria for individual parameters can be combined by the usual arithmetic operations as well as by the basic logical connectives AND ( 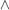 ) and OR ( 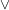 ). Conditions for individual parameters can be imposed by the binary modulo operation 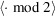 , facilitating the differentiation between two states by means of a mathematical case distinction between even and odd parameter values. In a graphical representation of a decorated twin structure, the different states are expediently depicted by means of different disc colours, radii or filling styles (solid or open).

A multitude of choices can be made, of which some represent a kind of differentiation exhibiting a chemically meaningful counterpart in an actual crystal structure (naturally, these are more interesting within a crystallographic context), while others lack such a correspondence. In practice, the different mathematical case distinctions can be associated with different chemical or physical states, such as representing a pair of distinct atom types (chemical colouring), or, alternatively or simultaneously, signed atom displacements ±*z* in the perpendicular out-of-plane direction (geometric colouring).

**Figure 4:**
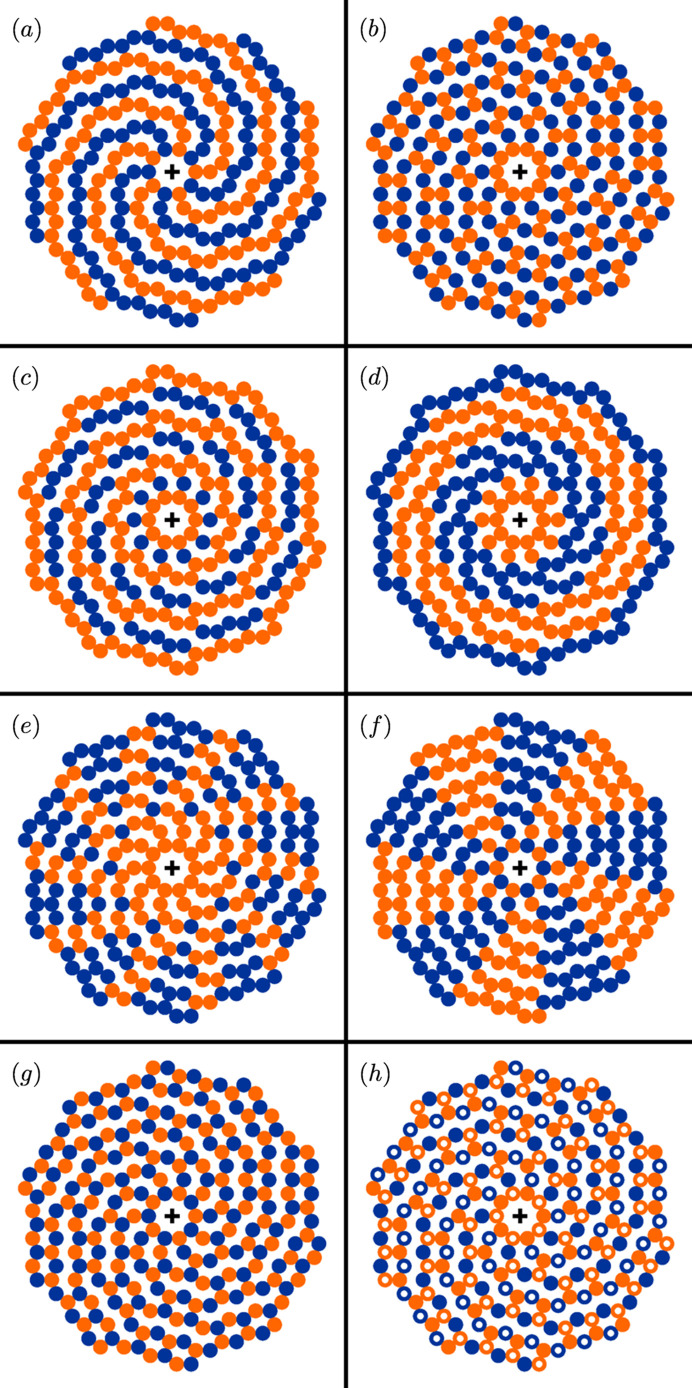
Binary decorations of chiral spiral cyclic twins. Conditions are represented as 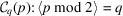 , where *q* ∈ {0, 1}. The symbols 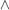 and 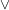 denote the logical AND and OR operators. Shown are the following cases: (*a*) alternating colours along *k*, condition 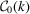 ; (*b*) alternating colours along ℓ, 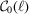 ; (*c*) alternating colours along ℓ in groups of the same baseline level, 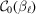 ; (*d*) alternating colours along ℓ in groups of the same *t* parameter, 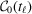 ; (*e*) highlighting *cis* configurations; 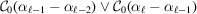 ; (*f*) highlighting twin domains, 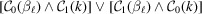 ; (*g*) alternating colours in *k* and ℓ combined, 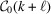 ; (*h*) alternating colours in ℓ and alternating filling styles in *k* and ℓ combined (case of NiZr), 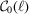 and 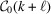 .

Arguably, the simplest case is to distinguish every other spiral branch from one another, in its entirety, by choosing one out of two colours [Fig. 4 ▸(*a*)]. Alternatively, one can alternate the colouring while going along the nodes of a spiral branch, making use of the different periods naturally occurring in the inclination pattern [Figs. 4 ▸(*b*) to 4 ▸(*d*)]. Using more refined combinations of conditions one can highlight the *cis*-configured nodes forming the twin boundaries [Fig. 4 ▸(*e*)] or the alternating twin domains in their entirety [Fig. 4 ▸(*f*)]. In a more complicated scheme one can also combine conditions on different attributes [Fig. 4 ▸(*g*)]: here, both the disc colour and its filling style alternate in unison along the spiral branches (orange solid versus blue open), but only the latter alternates among the spiral branches (orange solid versus orange open). This case represents the ideal tenfold twin structure of NiZr (Hornfeck, 2018 ▸, 2022 ▸; Hornfeck *et al.*, 2018 ▸), in which the colours are associated with the atom types and the filling styles are associated with the atom displacements ±*z*.

### 3.3. Exceptional cases: regular and aperiodic tilings

**Figure 5:**
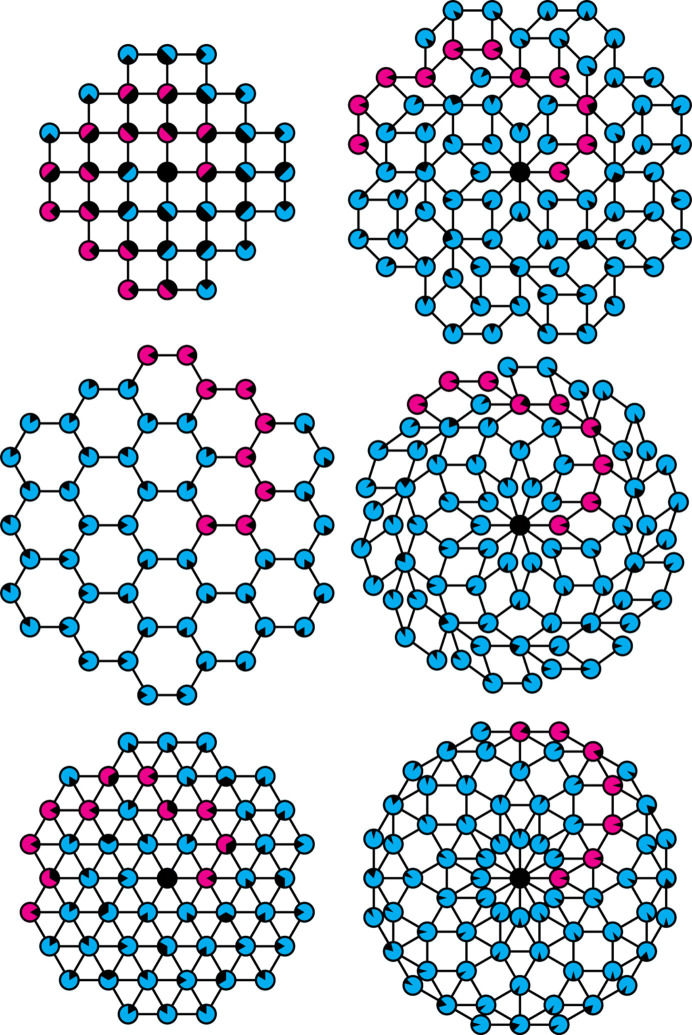
Exceptional cases, mostly of type (*m*, μ) = (2*q*, *q* − 1) for *q* > 1. Shown are the cases (top left) (4, 1), regular square tiling, (middle left) (6, 1), regular hexagon tiling, (bottom left) (6, 2), regular triangle tiling, (top right) (8, 3), Ammann–Beenker tiling, (middle right) (10, 4), generalized rhombic Penrose tiling, and (bottom right) (12, 5), Stampfli tiling. Individual spiral nodes are marked by black sectors, with each distinct sector orientation corresponding to a certain spiral branch, so that overlapping nodes stand out by containing more than one sector. See also Fig. 6 for a comparison of the chiral spiral cyclic twin tilings with their quasicrystal counterparts.

As the parameter ρ_*m*,μ_ is identified with the circumradius of the innermost regular polygon of the twin structure, it is naturally limited to values of ρ_*m*,μ_ > 0 since, for the limiting cases in which ρ_*m*,μ_ = 0, all of the first nodes Ω_*k*, 0_ as well as some of the subsequent nodes Ω_*k*,ℓ_ of adjacent spiral branches coincide, making the general model less elegant. Nevertheless, the resulting point patterns constitute interesting special cases, if one identifies the points with the vertices of a tiling. For the types (*m*, μ) = (4, 1) and (6, 2) this results in the regular square and triangular tilings, nicely corresponding with the type (6, 1) representing the regular hexagonal tiling, thereby complementing the set of possible regular tilings in two-dimensional space (Fig. 5 ▸, left). While type (6, 1) is not afflicted by a partial overlap of spiral nodes, it still constitutes a unique degenerate case amongst the chiral spiral cyclic twins insofar as it is, in fact, neither chiral nor twinned, since no perceptible twin boundary between the twin domains exists.

**Figure 6:**
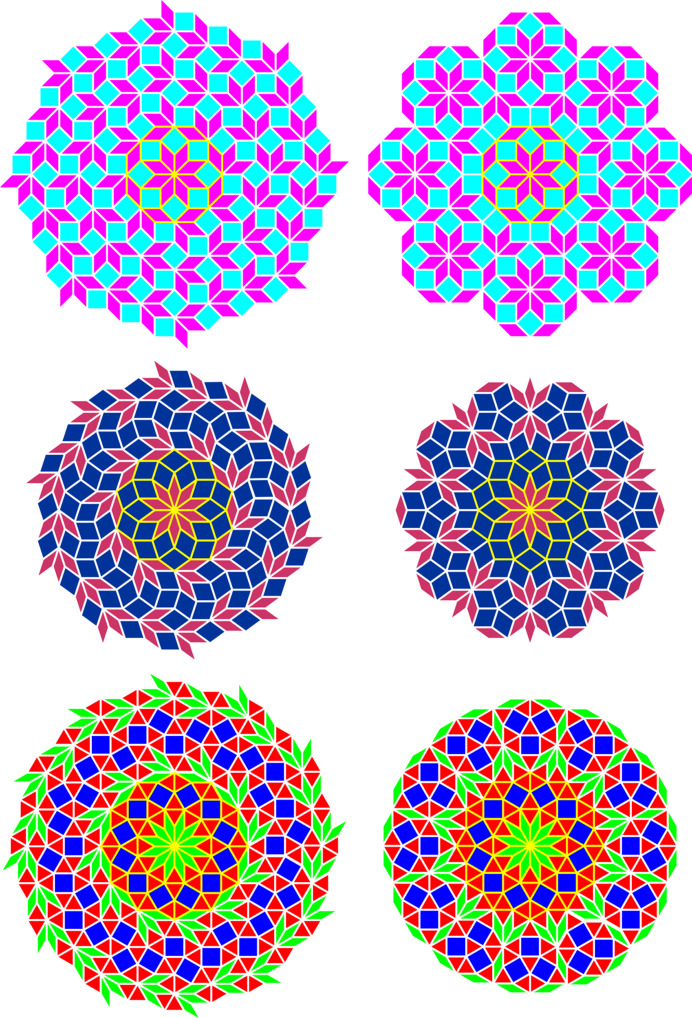
Comparison of tilings derived from the vertex set of the chiral spiral cyclic twins with *m* = 8, 10, 12 (Fig. 5 ▸, right-hand side from top to bottom) and the corresponding quasicrystal tilings constructed from the same set of tiles (triangles, squares, rhombuses). In particular, the case *m* = 8 is shown with cyan/magenta tiles (top), the case *m* = 10 is shown with dark-red/dark-blue tiles (middle) and the case *m* = 12 is shown with red/green/blue tiles (bottom). The chiral spiral cyclic twin tilings are shown on the left-hand side and the quasicrystal tilings are on the right-hand side. Note that in all cases each pair of tilings of common twin modulus *m* shares the same tile pattern at its core (highlighted by yellow lines surrounding the tiles). Note also that the quasicrystal tilings shown here are mirror symmetric with two-dimensional point group *m*mm (with the first letter ‘m’ in italic font type corresponding to the *m*-fold rotation symmetry, while the following letters ‘m’ in roman font each denote a mirror plane), while the chiral spiral cyclic twin tilings are chiral with the symmetry-reduced two-dimensional point group *m*.

In a similar fashion, types (*m*, μ) = (8, 3), (10, 4) and (12, 5) exhibit a perfect overlap for some of their spiral nodes. Instead of regular tilings, they form aperiodic tilings composed of different sets of rhombuses (Fig. 5 ▸, right). In the case of type (8, 3), the prototile set consists of a 45–135° rhombus together with a square, which is the same set as appears in the quasiperiodic Ammann–Beenker tiling of octagonal symmetry (Ammann *et al.*, 1992 ▸; Beenker, 1982 ▸). In the case of type (10, 4), the prototile set consists of a thin 36–144° rhombus together with a thick 72–108° rhombus, which is the same set as appears in the quasiperiodic rhombic Penrose tiling of decagonal symmetry; to be precise, a generalized Penrose tiling (Gähler *et al.*, 1994 ▸; Kari & Lutfalla, 2023 ▸), because a central patch of ten thin rhombuses, of vertex configuration ST (Zobetz & Preisinger, 1990 ▸), is not an allowed vertex configuration according to the aperiodicity-enforcing matching rules of the original Penrose tiling of thin and thick rhombuses. In the case of type (12, 5), the prototile set consists of a 30–150° rhombus together with an equilateral triangle and a square, which is the same set as appears in one of the quasiperiodic Stampfli tilings of dodecagonal symmetry (Schaad & Stampfli, 2021 ▸). Thus, all cases exhibit an unexpected and intriguing relation to their corresponding *m*-fold axial quasicrystals, although by their construction they still represent *m*-fold cyclic twins and hence are not truly quasiperiodic [Fig. 6 ▸; for many more examples of infinite tilings by rhombs, many of them aperiodic with *m*-fold rotational symmetry, see Schoen (2023 ▸)]. Similar rhombic tilings are obtained for types (*m*, μ) = (2*q*, *q* − 1) for *q* > 6.

Note that the same mathematical model yields both kinds of patterns just by a systematic change in parameter values.

## 4. Features of the general model

### 4.1. Algorithmic pattern formation

**Figure 7:**
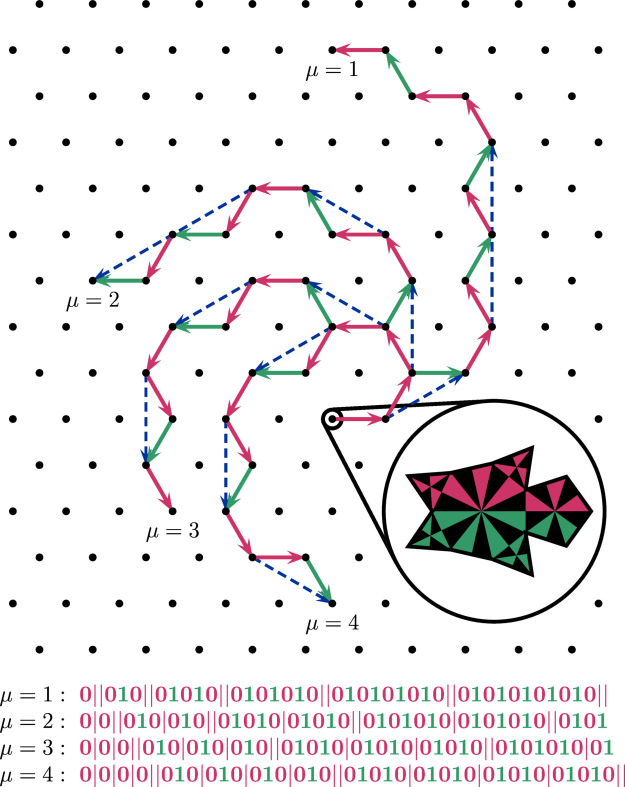
Triangular lattice paths representing the first 12 steps of the binary inclination sequences [alternating functions α, see equation (7)] resulting from the parameter values μ = 1 to μ = 4. The binary inclination sequences specify the relative directions (zero = red arrows = 2π/6, one = green arrows = −2π/6) in which a turtle has to turn for its next step on the lattice. Note that while the very first step is in an arbitrary direction (here, it is the rightward horizontal direction) it fixes the direction relative to which the next step proceeds, and by this all following steps in a recursive manner. Note that the same binary inclination sequences define a self-avoiding walk on their respective 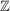 module, which is not true in the case of the triangular lattice (the reader might imagine the case μ = 5 to find the first counterexample). Note also that the definition of a spiral self-avoiding walk on a triangular lattice as used in the literature is different from our models, at least in a strict sense, unless one treats successive alternating steps as quasi-straight steps (dashed blue arrows), since direct straight steps are not allowed in our models otherwise. The binary inclination sequences are given for the first 36 steps, with single and double bars highlighting the pattern of μ times repeating, perfectly alternating, binary maximal subsequences of odd length.

The evolution process of a single spiral branch, as encoded in the spiral’s inclination sequence, resembles a case of turtle graphics. Here, a computational entity, the eponymous turtle, moves around the plane in a discrete step-by-step fashion, only relying on some internal information about its current location and its future movement relative to it, *e.g.* following movement instructions from a finite set of potential choices differing in their relative orientation (say, leftward or rightward, as a binary choice). The nature of the turtle’s movement is that of a non-random spiral self-avoiding walk on some lattice (Privman, 1983 ▸; Lin, 1985 ▸; Joyce & Brak, 1985 ▸; Seitz & Klein, 1992 ▸) or, generalized, a 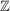 module [see Hornfeck (2022 ▸) for the definition]. In our case, the inclination sequence acts as a predetermined program set out for a turtle’s spiralling movement into infinity, yielding just the right combination of alternating steps, coming in pairs and thereby defining a straight movement, intermingled with the occasional one step more in the same direction, which guarantees the creation of the spiral’s curvature (Fig. 7 ▸).

Notably, the turtle’s reliance on relative movement about its current location emphasizes the mathematical property of *recursion*, in that each future location homed in on becomes updated to a current location, from which the same procedure repeats. In particular, this notion of recursion highlights relations to the algorithmic generation of patterns, to their coordinate-free description, to cellular automata (Reiter, 2010 ▸) and to Lindenmayer systems (Prusinkiewicz, 2000 ▸). The use of relative internal local information also connects the model to physical models of crystal growth, since atoms as entities without free will and memory can also only adhere to physical rules relying on this kind of information.

### 4.2. Crystallography and crystal chemistry

The precise arrangement of spiral nodes induces a locally periodic structure within the twin domains, exhibiting the following crystallographic and crystallochemical features.

The periodicity of the twin domains is described by a rectangular unit cell of lattice parameters *a* and *b*. The lattice parameter *a* is determined by the Euclidean distance between two unit steps inclined along the spiral. By simple geometric considerations, taking into account the inclination angle φ = ±2π/*m*, one finds *a* = 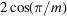 . Since the modulus *m* fixes the angle between adjacent twin domains, and this angle is the same as the inclination angle just mentioned, this also fixes the value of *b*, namely to *b* = 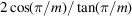 . Hence, for their axial ratio, 

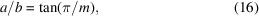

in accordance with a twin angle of φ = 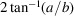 = 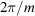 .

**Figure 8:**
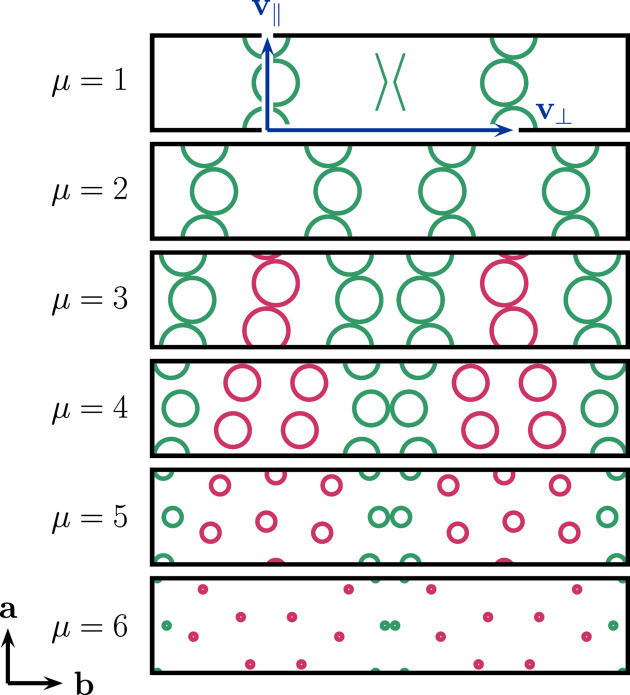
Twin domain unit cells for twins of modulus *m* = 16 with an axial ratio of *a*/*b* ≃ 0.2. Shown from top to bottom are the cases μ = 1 to μ = 6. The radius of the nodes is adjusted according to the minimal distance between closest nodes. Intraspiral contacts occur up to μ = 3 inclusive, while (partial) interspiral contacts prevail afterwards. Green nodes are symmetric under both a vertical and horizontal mirror plane, with unit cells containing only green nodes belonging to plane-group type *c*2*mm* (No. 9), while red ones are only symmetric under a horizontal one, with all unit cells containing them belonging to plane-group type *cm* (No. 5). A chevron motif 〉〈 resembling the arrangement of the green nodes is overlaid for the case μ = 1, together with the outlined parallel (intraspiral) and perpendicular (interspiral) vectors (blue) used in its definition (Fig. 2 ▸).

Within one unit cell one finds *N* = μ × 4 spiral nodes. Thus, the Pearson symbol for the corresponding three-dimensional crystal structure is *oSN*, denoting an orthorhombic side-centred unit cell (usually chosen to be a *C*-centred one). The nodes form a chevron-like pattern, naturally arising from the alternating inclinations, denoted 〉〈 for the case μ = 1 and 〉〉〈〈 for the case μ = 2. Using these shorthand symbols it is assumed that the **a** and **b** axes run along the vertical and horizontal directions, respectively. Going horizontally across adjacent unit cells the chevron pattern combines into a hexagon, symbolically 〈〉, a regular hexagon, in fact, for the special case (*m*, μ) = (6, 1). For the cases μ > 2 the chevron-like pattern becomes more complicated, in that intermediate chevrons are variably shifted along the **a** direction relative to those located about *y* ≃ 0 and *y* ≃ 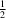 . This is indicated in the notation by using a left or right ceiling symbol, in order to highlight the mirror symmetry present and preserved with respect to the **b** direction (mirror line along *x*), yet absent due to symmetry breaking with respect to the **a** direction (mirror line along *y*). Thus, for μ > 2 one observes the pattern 〉⌉_μ−2_〉〈⌈_μ−2_〈, in which the subscript denotes the number of repeats of the intermediate chevrons (Fig. 8 ▸).

Thus, the symmetry of the twin domains can be described either by the plane-group type *c*2*mm* (No. 9; cases μ = 1 and μ = 2) or *cm* (No. 5; cases μ > 2). In this sense, there is a fundamental difference between the former and latter twins. The symmetry reduction can be described in a group–subgroup scheme (Müller, 2013 ▸) as being *translationengleich* of index 2: 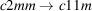 = *cm*.

In order to find possible three-dimensional crystal structures one can study the symmetry of special projections along the unit-cell axes of the orthorhombic space-group types, matching the aforementioned plane-group types. Together with the Pearson symbol and the axial ratio this allows for a systematic and targeted search in a database of crystal structures, such as Pearson’s Crystal Data (Villars & Cenzual, 2020 ▸). As before, the case (*m*, μ) = (10, 2) had been identified with the CrB (also known as the TlI) structure type of binary NiZr (Hornfeck *et al.*, 2018 ▸; Hornfeck, 2018 ▸). Because of the variability of its axial ratio *a*/*b*, the CrB structure type, with more than 300 distinct binary representatives known so far, allows for values of *m* ranging between about *m* = 8 and *m* = 10. Indeed, electron backscatter diffraction experiments hint at the occurrence of corresponding cyclic twins for the binary compounds NiB (*m* = 8) and NiGd (*m* = 9) (Niersbach, 2023 ▸; Niersbach *et al.*, 2023 ▸). A similar situation occurs for the μ = 1 case. Here, the structure types of α-U (unary) and Cu_0.75_Ti_0.25_ (binary, yet on one mixed occupied site) match the criteria, again allowing for a varying value of *m* for different compounds by virtue of a varying axial ratio. In particular, one finds (*m*, μ) = (6, 1) for the low-temperature modification of elemental Dy, as well as for binary Ag_0.6_Hg_0.4_ and Zn_0.75_Hg_0.25_, (*m*, μ) = (7, 1) for elemental α-U itself, and (*m*, μ) = (10, 1) for a metastable modification of binary Ga_0.9_In_0.1_. However, so far nothing appears to be known about cyclic twins with the respective rotational symmetry in these cases. Interestingly, in both cases the same space-group type is adopted, namely *Cmcm* (No. 63) with Wyckoff position 4*c* 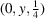 occupied once and twice for the α-U and CrB structure types, respectively. A structure type of the same symmetry, but with Wyckoff position 4*c* occupied three times and chevron pattern 〉〉〉〈〈〈, is given by binary ZrSi_2_ with (*m*, μ) = (12–13, 3). Structure types with this chevron pattern or higher-order ones are not considered further here, however, due to the aforementioned symmetry breaking precluding these ideal arrangements.

Finally, it should be emphasized that chiral spiral cyclic twins make up increasingly less plausible candidates for actual twinned crystal structures for increasing values of the parameter μ, since at some point the minimal interatomic distance becomes unreasonably small. The minimal interspiral distance is a function of *m* and μ, namely 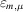 = 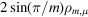 , and eventually becomes smaller than the fixed minimal intraspiral distance *d* = 1.

### 4.3. Continuous description

The *m*-fold chiral spiral cyclic twins are defined as a union of *m* symmetry-equivalent discrete spirals. Other notable discrete spirals are Ulam’s spiral arrangement of the prime numbers (Stein *et al.*, 1964 ▸), Vogel’s model of the sunflower head (Vogel, 1979 ▸; see Section 4.4), based on Fermat’s parabolic spiral as its underlying continuous curve, and, in particular, the spiral of Theodorus known since antiquity [Gronau, 2004*a* ▸; for a generalization see Crilly (2020 ▸)].

The spiral of Theodorus is also known as the Pythagorean spiral or *Quadratwurzelschnecke* [literally meaning square root snail in German and studied in the context of uniform distribution by Hlawka (1980 ▸)], emphasizing its construction from contiguous right triangles of side lengths in the ratio (*n*)^1/2^:1:(*n* + 1)^1/2^ with *n* being a natural number. It asymptotically approximates the likewise famous Archimedean spiral and has an interpolation formula due to the systematic approach of Davis (1993 ▸), which is extensively discussed in the context of analytic number theory (Gautschi, 2010 ▸) and can be generalized to other spirals (Gronau, 2004*b* ▸).

**Figure 9:**
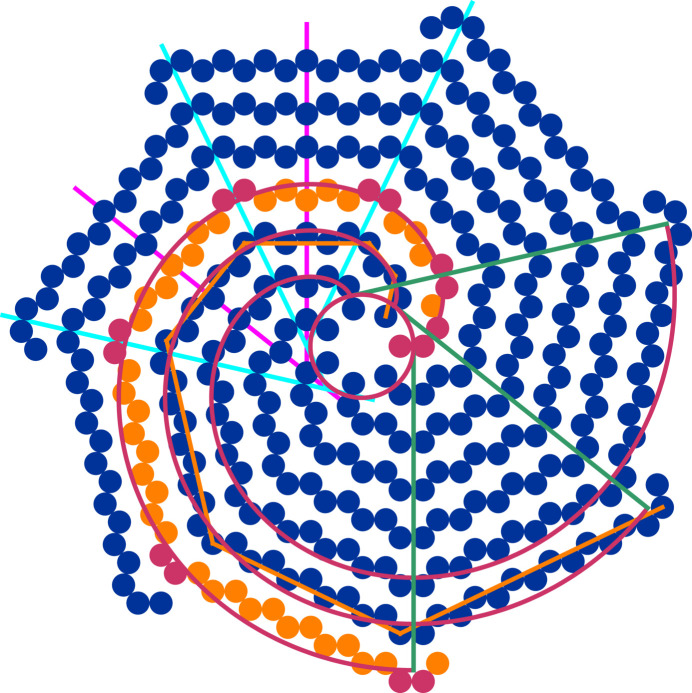
A chiral spiral cyclic twin with *m* = 7 and μ = 1. Shown are 7 × 52 = 364 nodes (mostly represented by blue discs of unit diameter). The spiral branch at *k* = 0 is highlighted with respect to its alternating all-*trans* configured segments (orange discs) and its pattern-breaking *cis*-configured corners (red discs), conferring curvature to the spiral branch. Overlaid are three out of seven equally spaced half-involute spirals originating from a base circle of radius *R*_I_ (red circle and spirals at *k* = 0, 1, 2) forming a double orthogonal system with three out of seven equally spaced half-lines (green lines). The spiral branch at *k* = 1 shows a rectified curve (orange polygonal curve or spirangle) used for the estimation of a spiral branch’s arc length in relation to that of its corresponding circle involute. Note that *R*_I_ does not yield a perfectly symmetric separation of the twin boundaries (green lines), for which a slightly smaller radius *R*_B_ proves to be better suited (cyan lines). A third radius *R*_C_ is associated with the perfect delineation of the twin centres (magenta lines).

Naturally, knowledge of such an interpolation formula for the case of chiral spiral cyclic twins would be very pleasing. It has been shown before (Hornfeck, 2022 ▸) that there does indeed exist a relation to continuous curves, namely involutes of the circle. These spirals are characterized by the unique geometric property of having a constant orthogonal distance of magnitude 2π*R*_I_ between successive turns of the curve, *R*_I_ being the radius of the associated base circle. Moreover, families of circle involutes form a double orthogonal system with families of their generatrices. Both families can be neatly given by a complex parameterization (of parameter *t*), namely 

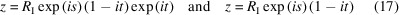

for the circle involutes and the generatrices, respectively (Ferréol, 2023 ▸). Here, *s* denotes the common starting point of an involute and its associated generatrix. The generatrices are straight-line segments (half lines) which can be identified approximately with the boundaries between twin domains (Fig. 9 ▸).

Unfortunately, there is no simple polar equation of the form ρ(θ) known for the circle involute, from which *R*_I_ can be determined. However, the polar equation of an Archimedean spiral that is asymptotic to a circle involute starting at the point (*R*_I_, 0) and for θ → ∞ is given as the linear equation ρ(θ) = *R*_A_(θ + π/2). Note that ρ(−π/2) = 0, thus, in a parametric plot, the Archimedean spiral starts at the origin (0, 0) and proceeds in the direction of the negative *y* axis, and hence is rotated clockwise by 90° relative to the circle involute [compare Fig. 8 in Hornfeck (2022 ▸)]. Accordingly, in a polar plot, there is a non-vanishing intercept with the *y* axis equal to *R*_A_(π/2).

The least-squares problem for a spiral dataset consisting of *N* points can thus be stated as ρ_*i*_ = *R*_A_θ_*i*_ + *R*_A_(π/2) + ɛ_*i*_, with ɛ_*i*_ forming the error terms in the standard least-squares sum 

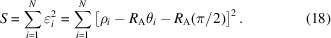

As usual, to find the optimum *R*_A_ one proceeds with calculating the first derivative d*S*/d*R*_A_, yielding 

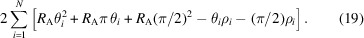

Setting this equal to zero and solving for *R*_A_ yields 

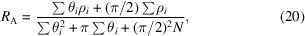

with all summations to be understood as covering the range from *i* = 1 to *N*. Finally, the following identifications are made (the index *i* corresponding to the index ℓ): 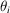 = 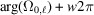 and ρ_*i*_ = |Ω_0,ℓ_|. Here, the value of θ_*i*_ is the reconstructed value of the angle, taking into account increments *w*2π according to the winding number *w* of the spiral about its origin.

While a least-squares fitting approach to determine *R*_A_ ≃ *R*_I_ always works, a merely numerical solution is always less satisfying than an analytical, even if only an approximately valid one. To obtain an example for the latter, one can proceed as follows. The arc length *S*_I_ of a circle involute is given as *S*_I_ = (1/2)*R*_I_ *t* ^2^, from which its base circle radius can be determined as *R*_I_ = 2*S*_I_/*t* ^2^. Now, for a fixed value of the parameter *t* the unknown arc length of the circle involute has to be of a similar length to the arc length of the discrete spiral of a given chiral spiral cyclic twin, *S*_I_ ≃ *S*_CSCT_, the latter of which can be graphically determined from the number of nodes and the lengths between them as occurring up to the chosen value of the parameter *t*. For our purposes we take *t* as an integer multiple of a full angle, *t* = *w* × 2π, in which *w* denotes the winding number. Note that a full turn in terms of the parameter *t* does not correspond to a full turn of the circle involute’s traced out trajectory. Instead, one has to count the discrete spiral’s nodes up to 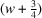 turns for a proper estimation of its arc length. Also, the trivial estimate of the arc length, based on unit distances between nearest nodes, does not yield a good approximation to the continuous spiral, as can easily be confirmed from a plot of both together. A more refined, and much better, estimate can be obtained by considering the slightly more rectified next-nearest node distances of 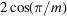 , occurring *n*/2 times. This rectified spiral alternately traverses along the outer and inner points of the alternating segments, thereby yielding a more balanced estimate of the arc length 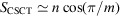 and the radius 

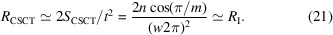

For general chiral spiral cyclic twins the number of nodes up to 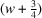 turns is *n* = *w* ^2^ *m* ^2^/μ. Thus, 

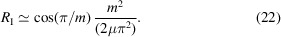

This approximation turns out to be precise within only a few percent of deviation with respect to the Archimedean spiral least-squares fitted values, generally overestimating *R*_I_. Interestingly, the circle involute’s radius thus determined and the innermost regular polygon’s circumradius are asymptotically equivalent, *R*_I_ ≃ ρ_*m*,μ_, for *m* → ∞. The term *m* ^2^/(2μπ^2^) is the base term in the Laurent series expansion of ρ_*m*,μ_ at *m* = ∞.

While it does establish the relation between the discrete and continuous descriptions, the involute base circle radius *R*_I_ does not yield the best description for the geometric locus of the twin boundaries. It is to be observed, though, that the centre lines of the twin domains trace out the edges of the regular *m*-gon of vertex set 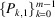 . Connecting the midpoints of its edges inscribes another regular *m*-gon, whose edges are traced out by the boundary lines of the twin domains. The pair of radii of the circles to which the centre and boundary lines are tangent, *R*_C_ and *R*_B_, are then given as the respective inradii (apothems) of the pair of regular *m*-gons, which can be obtained from their corresponding circumradii by multiplication with the factor 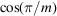 . In particular, the circumradius of the regular *m*-gon associated with the centre lines is given as ρ_*m*,μ_ + 1, while the circumradius of the regular *m*-gon associated with the boundary lines is equal to the former regular *m*-gon’s inradius. Thus, 

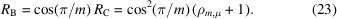

Then, the geometric locus of the centre and boundary lines of the twin domains is described by the complex parameterizations 

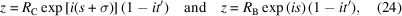

respectively, in which *s* = 2π*k*/*m*, σ = π/*m* and *t*′ = *t* − σ.

The continuous description highlights the fact that the central part of a chiral spiral cyclic twin forms a structure on its own, an inner aperiodic core domain distinct from the outer periodic twin domains originating from its periphery. This has been described as a quasicrystalline seed overcoming the nucleation barrier more easily than a crystalline one, yet eventually triggering the growth of a twinned crystal (Hornfeck *et al.*, 2018 ▸).

### 4.4. Packing densities

A natural question to ask about any spatial arrangement of points concerns the optimality of an associated circle packing. Since the distance *d* between two consecutive nodes along one spiral branch is set to unity, the radial extension of corresponding circles is bounded from above and cannot exceed *d*/2 = 0.5. However, for some combinations of parameters *m* and μ the distance *d* will not be the minimal distance of the whole point set. In these cases the minimal distance corresponds to the edge length of the innermost regular polygon: 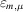 = 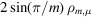 . Thus, choosing the radius according to 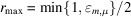 guarantees a pattern of equal-sized maximally extended non-overlapped circles.

The radius *R*_max_ of the reference circle used for the calculation of the circular packing density is now defined as the maximal absolute value found among all *n* nodes of one spiral branch plus the maximal radius of a single node: 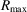 = 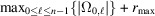 .

The packing density, as a function of the number of nodes *n* and for a given choice of the model parameters *m* and μ, is then given as 

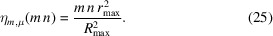




**Figure 10:**
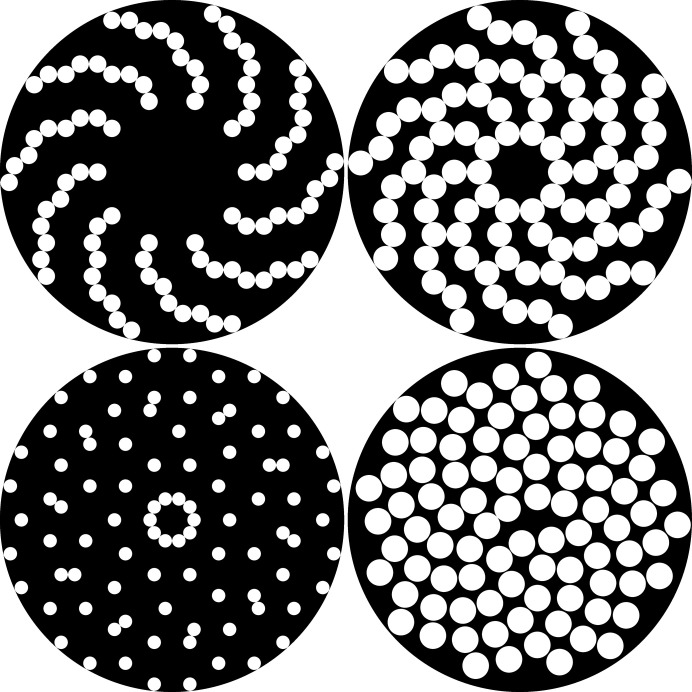
Circle packings for tenfold twins (*n* = 9, *m* = 10) of varying packing density η. Shown are the cases μ = 1 (top left, η = 0.234), μ = 2 (top right, η = 0.477) and μ = 3 (bottom left, η = 0.140), in comparison with a sunflower head consisting of 90 florets (bottom right, η = 0.546). The densest possible arrangement of 90 circles within a circle [not shown here, but depicted by Lubachevsky & Graham (1997 ▸)] has a packing density of η = 0.809.

As an example, Fig. 10 ▸ shows the circle packings for the cases (*m*, μ) = (10, 1), (10, 2) and (10, 3) of tenfold twins with a fixed number of *n* = 9 nodes along each spiral branch (the pattern thus comprising 90 nodes in total). The choice of *n* is rather arbitrary, in that a higher or lower number of circles would give a similar impression upon visual comparison, yet an optimal packing for this exact number is known, which aids as a reference. The circles are represented as white discs on a black disc background, visually highlighting the varying packing density as well as the different degrees of relative circle sizes.

For increasing values of μ the packing density runs through a maximal value of η_10,2_(90) = 0.477, which is remarkably high for this kind of twin structure, yet still far away from the maximum packing density of η_°_(90) = 0.809, as achieved by a finite packing of 90 circles within a circle (Lubachevsky & Graham, 1997 ▸). In comparison, both values are still considerably lower than the optimal packing density of η_tri_ = π/(12)^1/2^ ≃ 0.907, as observed for the closest packing of unit circles on the nodes of a triangular lattice in a two-dimensional plane (Tóth, 1942 ▸). It is also interesting to compare these values with the optimal packing density η_sun_ = 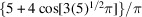 ≃ 0.817 found in another discrete two-dimensional spiral pattern, namely Vogel’s model of a sunflower head (Ridley, 1982 ▸), with its particularly elegant polar parameterization *r* = (*n*)^1/2^, θ = *n* × [3 − (5)^1/2^]π, the latter angle in radians corresponding to the golden angle equal to 137.508° (Vogel, 1979 ▸). However, for a finite patch consisting of 90 nodes, Vogel’s sunflower pattern has a comparable packing density of η_sun_(90) = 0.546 with respect to the (10, 2) case.

The packing density as defined in equation (25) can be approximated as 

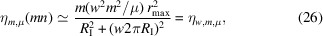

taking into account the value of *n* = *w* ^2^ *m* ^2^/μ and approximating the maximal radius by the involute base circle radius *R*_I_ [equation (22)] as 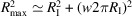 .

While this approximation fails to reproduce the numerical fluctuations in the exact values of the packing density η_*m*,μ_(*m* *n*), it facilitates the estimation of an asymptotic packing density for the case *n* → ∞, namely by using *w* as a proxy for *n*: 

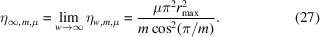

Now, for constant *m* and varying μ, the packing density 

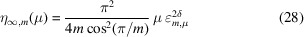

traverses through a *potential* maximum, corresponding to the crossover point of the two *r*_max_ regimes (at which ɛ_*m*,μ_ = 1 exactly), accompanied by an exponent change from δ = 0 to δ = 1 in equation (28). Solving ɛ_*m*,μ_ = 1 for μ, independent of any approximation, one obtains 

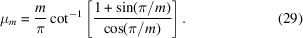

In fact, for all cases of interest (*m* > 4) μ_*m*_ follows the much simpler relation 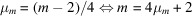 .

For a specific choice of *m*, however, the maximal packing density at the crossover point 

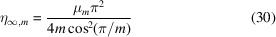

is only ever *realized* for the cases in which μ_*m*_ is an integer, corresponding to the existence of interspiral distances of unity.

Eventually, the maximal packing density in the double limit *n* → ∞ and *m* → ∞ is given as 

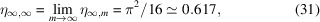

implying that, in summary, the chiral spiral cyclic twins represent, even at their best, rather loosely packed arrangements of discs, say, in comparison with Vogel’s sunflower spiral, the maximal value being about the same as the circle packing density for a regular hexagon tiling: η_hex_ = π/(27)^1/2^ ≃ 0.605 (Williams, 1979 ▸, p. 49).

### 4.5. Eigenvalue equation

All chiral spiral cyclic twins share the property of having a constant unit distance between the nodes along a single spiral branch. Now, an interesting question is, can the individual spiral branches be connected by a set of constant unit distances from the origin?

Translated into algebraic language, this asks whether the innermost regular *m*-gon’s circumcircle radius ρ_*m*,μ_ can be obtained as the absolute value of some finite weighted sum of the complex roots of unity, 

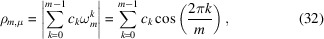

for some set of integer coefficients *c*_*k*_ and depending on the choice of the parameter μ. In particular, one looks for a minimal representation, optimal in having the least number of terms and a minimal value for the sum of coefficients *c*_*k*_.

This can be answered in the affirmative for certain cases. For instance, ρ_8,1_ = 1 + (2)^1/2^ can be represented as 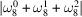 . An alternative choice is 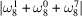 , in which the complex roots have been arranged symmetrically to the real axis of the complex plane. In fact, if one minimal solution exists, another *m* − 1 ones do, by means of cyclic permutation and as an algebraic expression of a twin’s *m*-fold rotational symmetry.

In fact, the question can be formally stated as an eigenvalue equation, namely as 

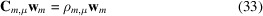

for the eigenvalue ρ_*m*,μ_ and the *m*×1 column eigenvector **w**_*m*_ = 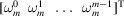 , where T denotes transposition and where the *m*×*m* eigenmatrix is given as 

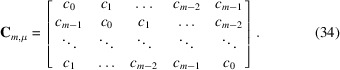

In fact, since the eigenvalue and eigenvector are given, and the eigenmatrix is sought, this is an instance of an inverse eigenvalue problem. The eigenmatrix **C**_*m*,μ_ has the special form of a circulant matrix, in which the columns and rows are cyclic permutations of one another. In this case it suffices to state the zeroth row vector **z**_*m*,μ_ = 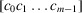 of the matrix in order to define all of it. For the example given above, the zeroth row vector is given as **z**_8,1_ = [11000001]. Some other solutions are: **z**_10,1_ = [1200000002], **z**_10,2_ = [0100000001], **z**_10,3_ = [0010000010], **z**_12,1_ = [320000000002] and **z**_12,2_ = [110000000001].

However, this is not true in general. In particular, a computer search for solutions with *m* being an odd number smaller than 12 remained futile in all possible instances, as was the case for *m* being an even number for the specific parameter pairs (*m*, μ) = (8, 2), (12, 3) and (12, 4).

**Figure 11:**
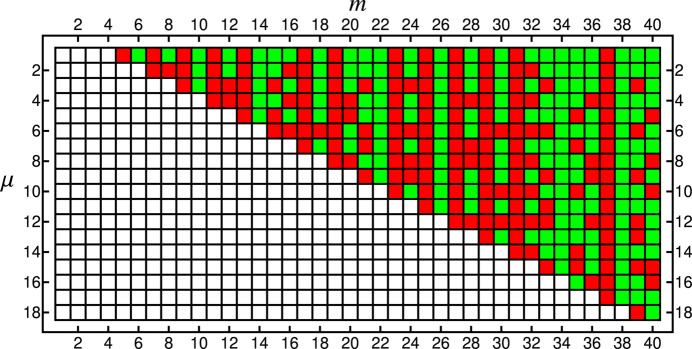
Existence of solutions to the eigenvalue problem stated in equation (33), in which ρ(*m*, μ) is the eigenvalue (shown for *m* ≤ 40). Cases for which a solution exists are marked by a green box, and those for which a solution does not exist are marked by a red one. White boxes represent non-admissible cases, for which ρ(*m*, μ) is (i) non-positive (in the case μ < *m*), (ii) non-definite (μ = *m*) or (iii) just repeats the already observed pattern (μ > *m*). This last case distinction reflects the fact that ρ(*m*, μ) = ρ(*m*, μ + *km*) for 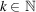 . Note the preferential absence or presence of solutions for *m* being an odd or even number, respectively.

The reason for this behaviour is rooted in the property of ρ(*m*, μ) of being, in general (that is for 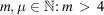 ), an algebraic number, yet not always an *algebraic integer*. An algebraic integer is a special case of an algebraic number, namely a root of a minimal polynomial in one variable, with integer coefficients and with the coefficient of highest degree being equal to unity (a monic polynomial). Now, since the cosine of an angle which is a rational multiple of 2π is an algebraic integer, and because the sum and product of algebraic integers are algebraic integers, the rightmost expression in equation (32) always is an algebraic integer, thereby requiring ρ(*m*, μ) to be the same in the case of equality. This property can be checked, for instance by using the built-in function AlgebraicIntegerQ in *Mathematica*. Those cases for which solutions exist (Fig. 11 ▸ shows cases up to *m* = 40 inclusive) are special in the way that all nodes of the chiral spiral cyclic twin pattern can be indexed by a tuple of up to *m* separate integers, thereby representing a connection to so-called 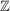 modules and the higher-dimensional crystallography of aperiodic crystals (Hornfeck, 2022 ▸).

## 5. Applications

The general model of chiral spiral cyclic twins developed in this work might find some applications outside its crystallographic realm due to the general importance of circle involutes in science and engineering, for which the chiral spiral cyclic twins constitute a discrete polygonal curve counterpart.

In the following the focus will be set to two application areas, each covering wide ranges of physical effects, material classes and engineering tasks, in which continuous circle involutes have been used, and which therefore might offer applications for discrete polygonal involutes as well. One might distinguish the use of continuous circle involutes in either (i) electromagnetically responsive nanostructures or (ii) microelectromechanical systems (MEMS) metamaterials. Here, one main distinguishing factor might be seen in the actual scale of the setup (nanometres to micrometres), and another one in whether the setup is regarded as being either a static or a dynamic one in mechanical terms. A third kind of difference is due to the use of either single or multiple spirals, the latter arrangements commonly called multifilar (Isik & Esselle, 2009*a* ▸,*b* ▸) and particularly akin to the case of chiral spiral cyclic twins.

In any case, a discrete spiral pattern might be the product of a suitable microscopic manufacturing process. Depending on all the details of the metamaterial’s construction, the interaction of it with respect to the electromagnetic field can be fine tuned, with applications being dominant in the visible part of the electromagnetic spectrum (*e.g.* Kerber *et al.*, 2018 ▸), yet sometimes extending into the tera- (Kan *et al.*, 2015 ▸) or gigahertz (Zito *et al.*, 2013 ▸) range. Besides optical uses of MEMS (Solgaard *et al.*, 2014 ▸), acoustic ones (Udvardi *et al.*, 2017 ▸) have also been described, in particular using discrete multi-spirals for phased array beam forming (Amaral *et al.*, 2018 ▸).

In many cases, unique physical effects and technologically important properties result from the aperiodicity and chirality specific to the two-dimensional metamaterial structure (Maciá, 2012 ▸; Dal Negro *et al.*, 2016 ▸). For instance, a device called a spiral axicon used in diffractive optics is able to create a wavefield with an orbital angular momentum (Jahns, 2017 ▸).

**Figure 12:**
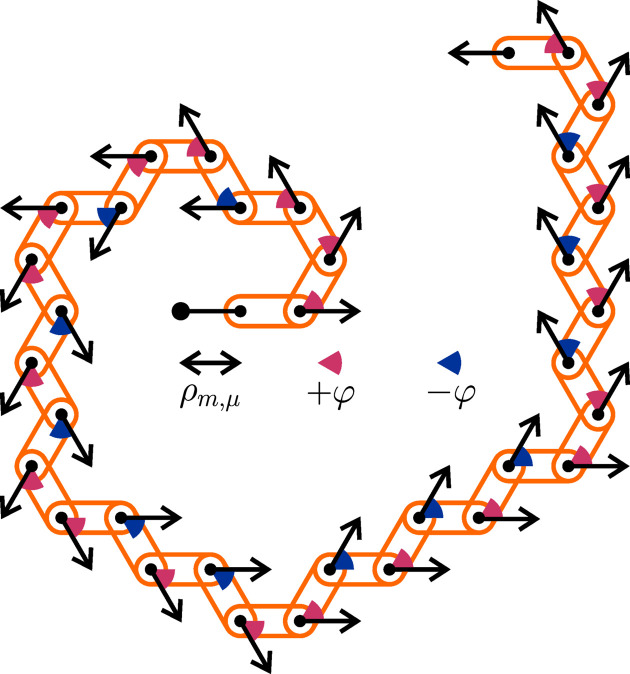
A schematic drawing of a mechanical model of a single spiral arm composed of interlocked fixed-length chain segments (orange), located at a variable distance of ρ_*m*,μ_ from a central node (large black dot) by a translational actuator (double arrow) and equipped with rotational actuators (single arrows with coloured circle sectors attached) at their common pivots (small black dots), by which they can be oriented relative to one another in fixed angular increments of opposite sign ±φ (here, φ = 2π/6; in general, φ = 2π/*m*). A full mechanical model would consist of *m* single spiral arms acting in unison within an *m*-fold rotational symmetric arrangement around a common centre (large black dot).

**Figure 13:**
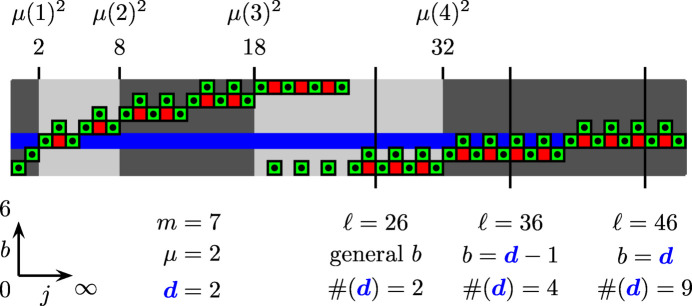
Counting the number of occurrences of the digit *d* in the modular finite inclination sequence 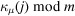 . Shown is the example for the inclination sequence 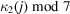 , a choice of digit *d* = 2, and the three distinct cases of general *b*, *b* = *d* − 1 and *b* = *d*. The baseline level *b* = 2 is highlighted by a blue horizontal bar on which the counting proceeds, while the distinct cases are highlighted by black vertical bars at different lengths ℓ of the sequence. For general *b* the baseline level does not coincide with any part of the inclination sequence, while for *b* = *d* − 1 and *b* = *d* the upper and lower parts are at the baseline level (the baseline function β is given by the segments connected by red boxes). The dark- and light-grey regions highlight different μ*t* ^2^ intervals.

One of the remarkable properties of the family of chiral spiral cyclic twins is the existence of distinct regular solutions, differing in their respective μ parameters, for the same twin modulus *m*. Since the geometric parameterization of the whole pattern depends only on the choice of circumradius ρ_*m*,μ_ and the relative pattern of angular inclinations, one can conceive the possibility of an actual mechanical model with radially movable spiral arms consisting of interlocked chain segments with the ability to rotate independently around each of their common pivots by means of local actuators (see Fig. 12 ▸ for a schematic presentation), thereby allowing for a mechanical transition between distinct (*m*, μ) states. This will be particularly interesting for designs of higher rotational symmetry (larger twin modulus *m* values), given their higher numbers of possible states (larger twin multiplier μ variety).

Finally, it might be interesting to combine a chiral spiral cyclic twin antenna/sensor array mounted on a rigidly foldable origami design of corresponding symmetry, namely those of the ‘flasher’ type, as originally devised by Jeremy Shafer [see Chen *et al.* (2019 ▸) for an application].

## 6. Conclusions

Compared with the previously described parameterization of chiral spiral cyclic twins in Part I of this work (Hornfeck, 2022 ▸), several improvements have been made:

(i) The general model presented here greatly extends the previous known cases of chiral spiral cyclic twins in terms of admissible values for the model’s integer parameters *m* and μ. In particular, it adds new representatives for all cases in which *m* is an odd number (Fig. 3 ▸). This encompasses some degenerate cases of remarkable regularity, including all regular periodic tilings of the plane, as well as cyclic twins related to some well known aperiodic tilings composed of rhombuses and squares (Figs. 4 ▸ and 5 ▸).

(ii) The parameterization has been simplified and streamlined. Now only one formula for the parameter previously denoted τ_*m*_ is needed, instead of two. As its geometric role as a radius is clear now, it is renamed as ρ_*m*,μ_ [equation (6)]. The essential results of the previous model are maintained, namely those for the cases (*m*, μ) = (8, 1), (10, 2) and (12, 2).

(iii) The baseline function β has been described within a more general and systematic scheme [equation (11)], clarifying the role of the parameter μ as fundamental period for all derived integer functions.

(iv) The interrelation between *m* and μ has been clarified, namely their mutually limiting character. Being strictly coupled previously, thereby creating a one-parameter family of twins, proved to be too strong a constraint. The improved model features more variety without imposing any *ad hoc* coupling.

(v) An alternative and more efficient way of summing the complex roots of unity [equation (3) and Appendix *A*2] has been introduced, based on the frequencies of occurrence of complex roots of unity.

(vi) Finally, the relation between discrete and continuous spirals has been made more precise by deriving a parameter-based approximation of the circle involute’s base circle radius *R*_I_ [equation (22)].

## Appendix A Combinatorics of the inclination sequence

### A1. Counting digits in the non-modular infinite sequence

If the inclination function κ(*j*) is taken modulo *m*, the resulting integers correspond to one of *m* directions as defined by the *m*th roots of unity, complex numbers *z* satisfying the cyclotomic equation *z* ^*m*^ = 1. However, due to its appearance in the phase part of the complex exponential and the cyclic periodicity associated with it, the modulo *m* operation can be omitted from the definition of the inclination function κ(*j*) as given in equation (7). In this case,  is the number of occurrences of a specific digit *d* > 0 in the infinite sequence  dependent on the choice of the parameter μ. Here, the exceptional case of *d* = 0 is defined to yield #_μ_(0) = 1 for all μ.

In order to find the number of occurrences #_μ_(*d*) of a given digit *d* in the string describing the inclination sequence for a given μ, one can separate its baseline part from its alternating one. The baseline part is determined by the rule that the digit *d* appears a period of  times. The alternating part is determined as an alternating sequence of zeroes and ones, sharing the same period *P*(*d*) and always starting with a zero. Since, by definition, the period *P*(*d*) = #_*d*_(0) + #_*d*_(1) is always an odd number, the number of zeroes and ones is given as

and

Since both sequences are added in an element-wise manner, the number of occurrences #_μ_(*d*) of a given digit is changed accordingly. Namely, for any given digit *d* the number of ones #_*d*−1_(1) on the previous level *d* − 1 increases its number of occurrences (positive sign), while the number of ones #_*d*_(1) on the same level *d* decreases its number of occurrences (negative sign): #_μ_(*d*) = *P*(*d*) + #_*d*−1_(1) − #_*d*_(1) = #_*d*−1_(1) + #_*d*_(0). This expression can be algebraically transformed and simplified according to equations (35) and (36) such that one finally obtains

Simple results exist for the special cases μ = 1 and μ = 2. A straightforward calculation yields #_1_(*d*) = 2*d*. Considering cases for even and odd *d* separately, and simplifying floor functions with integer summands in their argument on the way, gives #_2_(*d*) = *d* as a result. The general case is more complicated.

### A2. Counting digits in the modular finite sequence

The counting problem becomes much more intricate if one considers it for the modulo case in combination with a finite length ℓ of the inclination sequence.

As before, for reasons of clarity, almost all subscripts and function arguments have been dropped, with the functional dependencies on parameters and variables being derivable from the formulas.

Given a digit  and the length ℓ of the inclination sequence, one needs the following quantities and functions:

Here, β_ℓ_ is identical to the previously defined baseline function β(*j*) evaluated at the number *j* = ℓ, and *b* is the corresponding baseline function value after reduction modulo *m*. The parameter *t* going into β_ℓ_ is related to the supporting points μ*t* ^2^ of the baseline function and plays a role in the calculation of the partial period *p*_ℓ_. The parameter *Q* gives the maximum number of repeats to consider for the number *d* + *qm*, in which *q* denotes an index variable. The functions *P*(*x*) and *R*(*x*) are already making their appearance in the non-modular infinite counting case. The function *P*(*x*) determines the period of *x*, *i.e.* the number of repetitions of the baseline value associated with *x*. The function *R*(*x*) counts the number of occurrences of *x*, taking into account additive terms from the baseline level below and subtractive terms from the baseline level itself.

In order to count the number of occurrences of a given digit *d* in the modular finite inclination sequence  =  one has to consider three cases (Fig. 13 ▸), depending on the length ℓ of the sequence and on the derived baseline level *b*.

In the general case, in which neither *b* = *d* − 1 nor *b* = *d*, the number of occurrences will be given by the base counting term

in which the digit *d* and its equivalents modulo *m* up to a limit *Q* are represented as *D*_*q*_ = *d* + *qm* and  , respectively. In the special cases *b* = *d* − 1 or *b* = *d* one has to consider additional correction terms. The necessary case distinction is made by means of a modulo variant of the Kronecker delta  , which returns one if  and zero otherwise, thereby accounting for, as the maximum function in the definition of *R*(*x*) above, the special case *d* = 0 by preventing *d* − 1 from becoming negative. Then, we make the ansatz

The correction terms *C*_1_ and *C*_2_ turn out to be *C*_1_ =  and *C*_2_ =  =  . Here, *D*_*Q*_ = *d* + *Qm*. The form of these terms follows from considering partial periods *p*_ℓ_, either counting ones (floor function) or zeroes (ceiling function), as in the non-modular infinite counting case. In the case of the correction term *C*_2_ the full period *P*(*D*_*Q*_) has to be subtracted, since it was already fully accounted for in the base term *C*_0_ and occurs only partially when *b* = *d*. Altogether, this yields an alternative way of summation performed for each spiral node [equation (3)], having the advantage of needing only a constant number of terms.

### A3. The fundamental floor functions

In various instances of the aforementioned parameterization of the general model, expressions of the type  occur, in which *p* and *q* are integers, with *p* ≥ 0 being variable and *q* > 0 being fixed. The most prominent appearance is as  in the rule for the baseline function [equation (8)]. For distinct fixed values of μ and varying *j* one obtains different integer sequences  , namely infinite sequences of the non-negative integers repeated μ times. The associated *OEIS* (Sloane, 2023 ▸) identifiers for the first seven such sequences are: A001477 (μ = 1), A004526 (μ = 2), A002264 (μ = 3), A002265 (μ = 4), A002266 (μ = 5), A152467 (μ = 6) and A132270 (μ = 7). These integer sequences have the following common generating function (top) and associated series expansion (bottom):

Making use of the coefficient extraction functional [*x* ^*j*^], and by virtue of its shifting property, one obtains

A visual comparison of the exponents suggests that the values of the coefficients *p*_*j*_ are determined by the number of ways the number *j* − μ can be represented by the binary linear form *F*(*u*, *v*) = *u* + μ*v*, in which (*u*, *v*) is a pair of non-negative integer variables and (1, μ) are their associated non-zero, relatively prime, integer coefficients. For instance, in the case *j* = 23 and μ = 3 the derived number *j* − μ = 20 can be represented in seven distinct ways, namely as *F*(20, 0), *F*(17, 1), *F*(14, 2), *F*(11, 3), *F*(8, 4), *F*(5, 5) and *F*(2, 6). In general, the representations are given by *F*(*j* − μ, 0), *F*(*j* − 2μ, 1),  , *F*(*j* − *p*μ, *p* − 1), resulting in *p* distinct ways in total. The solutions can be graphically obtained as all of those points of a two-dimensional integer lattice which are located (and counted) on the line *v* = −(1/μ)*u* + (*j* − μ)/μ. They also can be interpreted in terms of a classical combinatorial coin change problem, with *j* − μ being equal to the total coin value and (1, μ) being the individual values of coins in infinite supply.

## Appendix B Deriving the general model

What follows is a more philosophical comment on the procedural pitfalls, helpful heuristics and tools of the trade in the derivation of the general model as presented in this work. It is meant to provide a qualitative glimpse into the sometimes erratic character of research, which is so often hidden from view.

The mathematical model is essentially based on two major parts, namely the integer inclination function κ(*j*) and the real circumradius ρ_*m*,μ_. Finding a meaningful parameterization for both has followed a meandering path, rich in at the time feasible but eventually false guesses, unveiling some near-misses on the way. Although mathematics is the most exact science known to man, it is not at all free of intrinsic ambiguities. For instance, these take the form of exact mathematical equivalences in which both sides of an equation, while being connected by an equals sign, might differ strongly in their physical meaning. This is exemplified by the existence of innumerable trigonometric identities [equation (6)], as well as by the algebraic equivalence of numbers in different representations or expansions. Again and again the question arises: What is the ‘best’ description featuring some intrinsic meaning?

This has been particularly true for the determination of the optimal circumradius ρ_*m*,μ_. This parameter plays a distinctive role among its kind. While the modulus *m* and the multiplier μ are integers, the circumradius derived from them is not (except for *m* = 6). As such, it has been more difficult to grasp in the theoretical development of the twin model. A numerical determination of any real number to an arbitrary specified precision is of course possible, by means of some kind of systematic search, yet this has turned out to be quite cumbersome in practice. Moreover, even a numerical determination to infinite precision cannot give any insights about its possible and plausible, yet empirically unknown, analytical expression. Naturally, this is an unsatisfactory state of affairs, challenging the conviction that patterns of such a high degree of regularity do not come without a similar regularity concerning the specific selection of their parameter values. All this can make finding patterns challenging, and for quite some time this stalled the discovery of the general model. Eventually a key insight, as described in Section 3, was obtained, yielding the sought-for closed-form expression.

In the course of the process some tools have been found to be indispensable: (i) Sloane’s *On-Line Encyclopedia of Integer Sequences* (*OEIS*; Sloane, 2023 ▸), (ii) Plouffe’s *Inverse Symbolic Calculator* (Plouffe, 1998 ▸), and (iii) both Wolfram’s *Mathematica* (Wolfram, 2013 ▸) and *Wolfram*|*Alpha* (Wolfram, 2023 ▸).
